# On the Treatment of Varicose Veins

**Published:** 1839-12-28

**Authors:** H. H. Smith

**Affiliations:** Paris


### MEDICAL EXAMINER.

DEVOTED TO MEDICINE, SURGERY, AND THE COLLATERAL SCIENCES.

No. 52.] PHILADELPHIA, SATURDAY, DECEMBER 28, 1839. [Vol. I.

### ON THE TREATMENT OF VARICOSE VEINS.

BY H. H. SMITH, M. D.

The numerous operations which have been attempted for the cure of varicose veins, from the time of Hippocrates up to the present day, have all had their supporters, and all been reported as more or less successful. No one period, however, has rested satisfied, but each succeeding age has given up the mode of its predecessors, in the hope of finding some other which might prevent the dangerous consequences of the phlebitis which followed their operations. Without intending, at present, to support any one plan of treatment, I here offer a few cases as illustrative of the opinions and practice of one who is certainly the most indefatigable of the French surgeons in the investigation of any thing connected with his profession. Although M. Velpeau's researches go back as far as 1830, yet he has not lost any of his interest on this subject, but is pursuing it, not in the belief that he can preserve the patient from a return of the disease, even when he succeeds in obliterating the enlarged vessels, but because "la necessité d'oblitérer les troncs veineux pour guerir les varices, etant bien établie, il fallait trouver le moyen le meilleur, le plus facile, et le moins dangereux, pour l'obtenir;"* and in one point he has certainly gained his object, that of a method *frequently* successful,—that is, not resulting constantly in serious consequences, or in the death of the patient, as out of more than a hundred cases operated on he met with no very dangerous symptoms, except a limited external phlebitis, phlegmonous inflammation, or small superficial abscesses.

The method was as follows:—The patient standing upright so as to distend the veins of his extremity, he seizes the largest of them, in a fold of the skin, and, elevating it, passes a strong common sized pin through the base of the fold, so as to allow the vein to rest with its posterior surface on the pin; then passing a strong waxed thread around the pin, (at first in the figure of 8, but latterly circularly,) he strangulates the tissues under the thread, pursuing the same course with the other principal varices, one above the other, to the number of three or four. Towards the sixth day after the operation the integuments slough and come away, leaving a small, superficial ulcer, generally easily healed.

Though obtaining considerable success, he at last was induced to change it, from the results of it on a man who entered the hospital of La Charité, aged thirty-four years, and in the enjoyment of perfect health, and on whom he operated the 4th of April, 1839. Every thing went well till the night of April 15th, when he was attacked with chills, vomiting, nausea, inflammation of the limb, &c.; and, on the 19th, fifteen days after the operation, died with all the symptoms of absorption of pus. At the autopsy the lungs were found healthy, but engorged,—the heart natural, but containing a fibriform clot,—the stomach filled with a greenish fluid, and the intestine contained a plaque, inflamed, and commencing to ulcerate. The left vena saphena, which had been operated on, contained grumous and fluid blood,—but the obliteration was not complete, even in the parts where the ligature had been applied. The right vena saphena was larger than common, and, as well as the vena cava, was filled with blood in a very fluid state. After this case he modified his operation; and, in September, 1839, tried it on the subjoined cases:

SALLE ST. FERDINAND.

*Case 1st.*—No. 6. Robin, aged thirty years, an ironmonger, entered the ward Sept. 6th, 1839, suffering from moderate varices of the right leg, which he has had for six years. Eighteen months since he received a slight blow on the front of the tibia of this leg, which produced an ulcer which has since remained open. It is now the size of a five franc piece, deep, and with indurated, shelving edges. These were touched once with nitrate of silver, poulticed for four days, and afterwards dressed by strips of adhesive plaster, very firmly applied over it, and removed only every three days.

*Sept. 12th.*—M. Velpeau operated in the following manner:—The patient being upright, he seized the vena saphena in a fold of the skin above the varices, about three inches below the knee, on the internal part of the calf of the leg, and with a *tenotome*, or sharp pointed, narrow lancet, made a small opening through the base of the fold, *behind* the vein, through which he passed an eyed probe, armed with a ligature. Withdrawing the probe on the outer side of the fold, he allowed the vein to drop and lie upon the ligature,—then introducing the probe by the same external opening, he passed it in front of the vein, between it and the integuments, bringing it out where it had entered on the inner side of the fold. He next passed a common pin *behind* the vein, in the course of the ligature, bringing its point through the loop formed on the outer side, where the ligature had been turned to pass in front of the vein,—then tightening the thread, he tied the ends around the head of the pin, so as to compress the vein upon the pin *under the skin*, by which he hoped to prevent one cause of danger, viz., the inflammation resulting from compression of the integuments, and completed the operation by cutting off the pin's point. One other ligature was placed

* Leçons Orales.

in like manner about three inches below the first, and just below the varix, so as to cut off all communication between the lower and upper part of the leg through the enlarged vessels. Considerable inflammation followed around the ligatures, but did not extend above the upper one, though the glands of the groin became a little enlarged. No dressing was applied, but the patient was confined to bed.

*Sept. 18th.*—The ligatures came away to-day; there is a slight ulceration immediately around the opening made for the introduction of the probe, and a few drops of pus followed the removal of the pins; poulticed.

*Sept. 21st.*—The inflammation has rapidly disappeared; the ulcer below has improved wonderfully; the shelving edges are gone; it has filled evenly from the bottom, and partially cicatrized; bandelettes continued to it; no application to the other parts.

*Sept. 26th.*—The veins are entirely obliterated, feeling hard and round like a nerve, and the circulation through them is obstructed; the cicatrization of the ulcer is very firm, and scarcely distinguishable from the surrounding parts; bandelettes continued, with the use of a bandage from the foot to the knee.

*Oct. 8th.*—The ulcer has healed; the patient has been walking about some days,—but there is no return of the circulation through the veins, which seem perfectly fibrous.

*Oct. 10th.*—Discharged cured twenty-eight days after the operation.

SALLE ST. AUGUSTIN.

*Case 2d.*—No. 43. Sermaise, aged sixty-two years, a sailor, has had extensive varicose veins for the last twenty-five years, and numerous ulcers on the leg for the same period, the first of which was produced by a burn.

*Sept. 9th.*—At present there is a deeply excavated, indolent ulcer, of near three inches square, and the leg presents the appearance common to all old ulcers. The veins are very much enlarged, and varicose from near the ankle to the middle of the thigh; he has also a hydrocele of the same side, and an inguinal hernia on the other, (right.) The ulcer was poulticed for two days, and afterwards dressed with strips of adhesive plaster, as in the other case.

*Sept. 15th.*—M. Velpeau placed two ligatures around the vena saphena on the thigh, about three inches apart, and the upper one about five inches from the groin, in the same manner as in the preceding operation. No dressing.

*Sept. 18th.*—Much inflammation has followed the operation, but it is confined to the vicinity of the ligatures; the vein between these points is tumid, and painful on the least pressure. He has slight fever, some little pain in the groin, but none in the abdomen. Poultice to the parts.

*Sept. 21st.*—The inflammation is subsiding, but the ligatures are yet firmly adherent. M. Velpeau to-day operated on the hydrocele with pure tincture of iodine.

*Sept. 24th.*—The last ligature came away to-day,—the other yesterday; there is a slight discharge of pus from the points of ligature, and the cutis has come off the inflamed portion, as in a blister, leaving the surface reddened and thick by the deposition of lymph in the subcutaneous cellular substance. Considerable inflammation has followed the operation on the hydrocele, but not more than is usual where the pure tincture is used of full strength. The patient is a little restless from this, with slight fever. The ulcer is much improved under the straps.

*October 3d.*—The inflammation over the vein has entirely subsided—the vein is obliterated, firm and hard down to near the knee; the parts around are yet thick and indurated.

*October 25th.*—The ulcer is nearly healed, with a firm cicatrix—bandelettes continued and a bandage to leg—patient walking about the ward, and the veins are yet firm and apparently impermeable.

*Nov. 1st.*—Discharged—well.

*Case 3d.—No.* 42.—Froucher, aged 27, hair-dresser, entered September 30th, with enlargement of the veins of the left testis, which has existed nearly eighteen months. The veins are moderately enlarged, and he has the usual symptoms of the disease.

*October 2d.*—M. Velpeau placed two ligatures around the largest of the veins, about two inches and a half from each other, and the highest about two inches from the inguinal ring. Ordered to remain in bed.

*October 4th.*—Very slight inflammation about the ligatures—slight pain in the veins between them—no pain along the cord or in the abdomen. No treatment.

*October 8th.*—More inflammation around the ligatures, but little at other points—ligatures are yet both firm.

*October 13th.*—One ligature came away to-day, with a slight discharge of pus from the opening; the other is still firm—inflammation disappearing, and the veins are thick and evidently contain a coagula—patient allowed to walk about with a suspensoir.

*October 17th.*—The second ligature fell to-day: the inflammation is trifling, but there is still a small ulcerated point around the last ligature—the veins are much firmer and more fibrous to the touch, and the patient leaves the ward to-day.

SALLE ST. FERDINAND.

*Case 4th.—No.* 14.—Denard, aged 33 years, a baker, of fine frame and robust health, has had varices of the right leg for the last ten years: there is and has been no ulcer. The veins are much enlarged, and tortuous, from the ankle to the knee—there are also two large plaques external to the course of the vena saphena, the largest of which is near two and a half inches in diameter, and situated on the middle and front of the calf—he is accustomed to stand the greater part of each night at his trade, and to press against his trough, a large portion of the time. Entered September 28th.

*Sept. 30th.*—M. Velpeau placed four ligatures around the veins, in the manner before mentioned—one on the saphena near the knee, above all the varices, one below them, near the lower third of the leg, and one above and below the large plaque, near the middle of the limb. No dressing.

*October* 3*d.*—Considerable inflammation and swelling around the ligatures, with much pain on touching the veins between them—no pain or inflammation above, nor is there pain in the groin. No treatment.

*October* 5*th.*—The inflammation has not spread upwards, but it has increased around the ligatures, which are yet firm: there is much heat and swelling at these points, and he cannot bear the slightest touch—ordered compresses wet in a decoction of quimauve (marsh mallows).

*October* 8*th.*—The lower ligature and the one at the lower part of the plaque came off to-day—pus followed their removal, and there is an ulcer, the size of a sixpence, at the point of the plaque: the parts are still inflamed, though rather less than at the last date: the veins are tumid, and, as well as the neighboring parts, tender to the touch. Poulticed.

*October* 9*th.*—At two o'clock last night he was attacked with rigors—slept none—has some cephalalgia—skin hot and dry—pulse 96—tongue furred—slight thirst—bowels not opened for two days—no inflammation above the knee is apparent, but the course of the vein and the groin is painful. Ordered V. S. ad f. ℥xvi.—poultice to parts, low diet, and eau de sedlitz.

*October* 11*th.*—The inflammation has increased around the points of the ligatures; the last two came away yesterday; there is well marked erysipelas in the leg, and also half way up the thigh; pulse 98, quick and irritated; skin hot and dry; cephalalgia; restless; sleeps little; tongue thickly furred but moist; anorexia; bowels opened yesterday; lymphatic glands enlarged; ordered a large poultice to the limb with mercurial frictions to the thigh.

*October* 13*th.*—Has much pain in the groin as well as in the lower part of the abdomen, and in the lower right side of the chest; coughs frequently, and expectorates thick frothy mucous; chest rather flat posteriorly on right side; slight bronchial respiration and crepitant rale; other symptoms augmented; ordered fifteen leeches to groin; lotion of *ferri sulphas* to thigh and poultice to leg; no other treatment.

*October* 14*th.*—Pulse 100, quick and feeble; skin hot; cough more troublesome; well marked pleuropneumonia of right side; expression languid; prostrated; at ten o'clock last night was attacked with violent pain in the right hypochondriac region; respiration quick and painful; thigh much inflamed and swelled; groin less painful; ordered fifteen leeches under the right mammary region; blister to thigh; poultice continued; ant. tart. gr. ss. in ℥iv. of syrup of gum.

*October* 15*th.*—Pulse 152, feeble and irregular; respiration quick and painful, 40 in the minute; cough very painful; sputa tenacious and brownish; less pain in chest since the leeches; prostration complete and evidently sinking; treatment continued.

*October* 16*th.*—Died at nine o'clock last night, seventeen days after the operation.

AUTOPSY.

*October* 17*th*—9 A. M.—Thirty-six hours after death.—No decomposition; general appearance natural; rigidity considerable; no œdema of extremities; chest flat on percussion on the right side; resonant on the left; slight cadaveric lividity of the back; blister on thigh and veins of right leg present the same appearance as during life; the skin on the right leg, being carefully dissected, showed the veins very much enlarged and varicose from just below the knee to the ankle, on the inner and front half of the leg. The vena saphena is very tortuous, frequently doubled on itself, and connected with a large plaque of veins on the front and inner part of the leg, anterior to its main trunk, and formed out of the enlargement of one of the superficial branches; a second and smaller plaque existed a little lower on the limb. At the upper and lower portion of the large plaque were two points of ulceration of the size of a shilling, with the destruction of a portion of the vein, corresponding to the points of the ligature. On the lower part of the saphena itself was a similar ulceration, and about the point of the upper ligature, near the knee, there was slight ulceration, with suppuration of the surrounding cellular substance. The lower point showed a destruction of near an eighth of an inch of the trunk of the vein, the extremity of which was at first thought to be obliterated; but subsequent maceration for twenty-four hours showed a small cavity in its centre, large enough to admit a needle. The upper portion of the vein, or the extremity above the upper ligature, admitted a fine probe, though it was very much thickened and diminished in calibre. The whole of the plaque was filled with pure straw-colored pus, and the vena saphena and a transverse superficial branch on the middle of the thigh contained more or less of the same. The whole course of the vein was marked by thickening of the cellular substance, and its coats were also much altered. The blood in the upper extremity of the saphena and the femoral was black and very fluid, and in the femoral, immediately at the entrance of the saphena, was a large clot, firmly adherent to its coats. The iliacs and the venæ cavæ were not altered, but contained the same dark fluid blood in a considerable quantity. The bowels and stomach were healthy, and the liver and kidneys were natural, and contained no abscesses; the spleen was more engorged than usual, but presented no other change.

*Chest.*—The right lung was inflamed, gorged with blood, and presented the appearance of the second stage of pneumonia: two large abscesses filled with pus were found in its middle lobe, near the surface. The pleura costalis and pulmonalis of this side was covered with lymph, and there was an effusion of near ℥viii. of serum, mixed with pus, in the pleural cavity. The left lung and pleura were healthy. The heart was natural, but the right auricle and ventricle contained a quantity of semi-coagulated blood; the left ventricle contained a large fibriform clot, adherent to the columnæ carneæ. *Brain*, healthy, but a little darker than usual.

*Case 5th.*—No. 20. Pachaud, aged 42, a cooper, has had enlargement of the veins of the left testis for fifteen years. They are much augmented and tortuous, forming folds, visible some feet from his person: the veins of the left leg are also varicose from the lower part to near the groin, forming great knots. Entered Octobe r8th, 1839.

*October 10th.*—M. Velpeau placed a ligature after the same method, around the veins above the greatest enlargement, and about one inch from the external ring. He also placed a second at the lower part of the scrotum, below the great mass of the varix; the tying of the ligatures caused considerable pain: this has not been the case to the same extent in the other patients; no treatment except to remain in bed.

*13th.*—Slight pain in testicle and along the cord; some fever; tongue slightly furred; bowels not opened for two days; little thirst; good appetite; ordered bleeding three palets: low diet and a suspensoir.

*14th.*—Has rested badly; slight cephalalgia and is irritable; skin hot, pulse 90, but regular; bowels opened by enema; cord inflamed, tumid and painful; left iliac and pubic regions also painful on pressure; scrotum inflamed, especially around the pins; ordered thirty leeches to cord and groin.

*15th.*—Slept little; less pain in groin; scrotum tumid from effusion into the cellular substance, so as to almost conceal the pins; cord less painful, but much enlarged; only ten leeches took, and did not bleed freely; erysipelas in scrotum; ordered thirty leeches again to same parts; poultice and diaphoretic mixture.

*16th.*—Leeches took well; less pain in parts; slight discharge from ligatures which were cut out to-day by M. Velpeau; other symptoms a little improved; ordered decoction of quimauve to parts, and a suspensoir.

*18th.*—Free discharge from ulceration of ligatures of a thin greenish pus, with a small portion of sloughing cellular substance; a small abscess has formed in the lower part of the scrotum, which was opened and gave exit to a sanious matter; slight cough with mucous rale; simple bronchitis; little fever, but is again restless; ordered laudanum, gtt. xxx. in ℥j. syrup of gum; half a dose morning and evening; poultice to parts.

*22d.*—General augmentation of symptoms since last day, except in cough, which is better: pulse 95, small and tense; skin hot and dry; thirst; anorexia; furred tongue; headach; no pain in epigastrium on pressure; scrotum more inflamed and enlarged; epididymis hard and painful; erysipelas has attacked the penis and cord; free discharge of sanious matter from points of ligature which show no disposition to heal; last night he had a heavy sweat, but says he had no chill; ordered mercurial frictions to parts, and a purgative enema; poultice continued.

*25th.*—Erysipelas disappearing; scrotum and parts less inflamed but still very tumid; less fever; bowels been opened freely; stools liquid and yellow; more cheerful; slight desire for food; treatment to parts continued.

*October 29th.*—The inflammation has disappeared, but the scrotum is yet twice the ordinary size and quite dense; little discharge from ulcers; veins thick and hard; penis and groin nearly natural; cord a little thickened; general symptoms good.

*November 2d.*—Left the ward to go to his family on account of some domestic troubles. The ulcerated points are not yet healed; the scrotum is smaller but still quite dense, and he has now no pain in the parts. The veins feel firm and are without doubt obliterated.

The result of this last case was much happier than was anticipated at one period from the symptoms; but the consequences in No. 14, also those of the case of a young Englishman resident in Paris, who died of a phlebitis resulting from Mons. Breschet's operation for varicocele, have caused many to regard these operations as far from safe or promising useful results. A late number of a British periodical also reports the death of a man in the hands of Mr. Cooper; three or four days after he operated on him by Mons. Velpeau's original method of the pins and figure of 8 ligature, external to the integuments. The autopsy of No. 14, and the opportunity of examining several cases of phlebitis and of the varices in patients dead of other diseases, have fully confirmed the pathology of the affection and the little benefit likely to result from any operation. In three cases of varices in the lower extremity, not only was the vena saphena enlarged, but the other vessels anastomosed, and were augmented so as to render the obstruction of the circulation almost impossible. But in order to produce this obliteration, be it by caustic externally; by incisions, or by ligatures, what change must take place in the vessel? The first is the determining of that degree of inflammation, which Cruvelhier has denominated *phlebite adhesive*, and which results in the effusion on the inner coat of a plastic matter which adheres strongly to its sides and obstructs the circulation, as it encroaches on the cavity of the vessel. By this obstruction, the blood moves more slowly; its thicker particles are deposited and attach themselves to this false membrane, until, by a gradual deposition, a very small canal is left in the centre, as found in the case of No. 14. If the inflammation stops here, a gradual contraction of the clot follows; its more fluid portion, and the colouring matter, are absorbed, and the vein becomes firmly closed, and like a fibrous cord, as shown in what was once the veins of the fœtus, but which by this change become a fibrous cord in the falciform ligament of the liver. But should the inflammation continue, the clot itself will become inflamed, pus will be deposited, not always in the centre, but often in its very substance. This, under favourable circumstances, may be absorbed and terminate in resolution, but if, as frequently happens, the inflammation proceeds, the proportion of the clot diminishes, that of the pus increases, the vein becomes filled with it, and its entrance into the circulation would be rapid, were it not that whilst the pus is forming, the inflammation has travelled up the vein, and gives rise to a second clot above, as shown in the clot found at the entrance of the saphena into the femoral in the above case. But this clot, when formed, was obliged to support the force of two powers, that acting on it from the saphena itself and that in the passage of the blood in the femoral, and in this way the pus having once reached the circulation in the large vessels, its distribution throughout the system must be so rapid as not to allow of the formation of a third clot. Even here, the formation of a clot is possible, as was shown in a case of metro-peritonitis, in which the iliacs and the lower part of the vena cava contained large clots, surrounded by a kind of membrane, and containing in their centre, a cavity large enough to admit a good sized probe. Supposing, therefore, a ligature applied to, or above a vein, beside the risks of the phlebitis, we should have to guard against inflammation of the subcutaneous cellular substance, and phlegmonous erysipelas, and after all, probably, have a recurrence of the disease from the increased circulation in the anastomosing branches, should we be so fortunate as to produce nothing more than an obliteration of the main trunk.

*Paris, November* 23, 1839.

